# Computed Tomography Assessment of Tidal Lung Overinflation in Domestic Cats Undergoing Pressure-Controlled Mechanical Ventilation During General Anesthesia

**DOI:** 10.3389/fvets.2022.842528

**Published:** 2022-04-01

**Authors:** Alessandro R. C. Martins, Aline M. Ambrósio, Denise T. Fantoni, Ana Carolina B. C. F. Pinto, Lenin Arturo Villamizar-Martinez, João H. N. Soares, Denise A. Otsuki, Luiz Marcelo S. Malbouisson

**Affiliations:** ^1^UFAPE Veterinary Intensive Care Unit, São Paulo, Brazil; ^2^Department of Surgery, School of Veterinary Medicine and Animal Science, University of São Paulo, São Paulo, Brazil; ^3^Department of Clinical Sciences, School of Veterinary Medicine, North Caroline State University, Raleigh, NC, United States; ^4^Department of Surgical and Radiological Sciences, School of Veterinary Medicine, University of California Davis, Davis, CA, United States; ^5^Discipline of Anesthesiology, Faculdade de Medicina da Universidade de São Paulo, São Paulo, Brazil

**Keywords:** computed tomography, pulmonary overinflation, ventilator-induced lung injury, tidal volume, controlled ventilation, cats

## Abstract

**Objective:**

This study aimed to evaluate lung overinflation at different airway inspiratory pressure levels using computed tomography in cats undergoing general anesthesia.

**Study Design:**

Prospective laboratory study.

**Animals:**

A group of 17 healthy male cats, aged 1.9–4.5 years and weighing 3.5 ± 0.5 kg.

**Methods:**

Seventeen adult male cats were ventilated in pressure-controlled mode with airway pressure stepwise increased from 5 to 15 cmH_2_O in 2 cmH_2_O steps every 5 min and then stepwise decreased. The respiratory rate was set at 15 movements per min and end-expiratory pressure at zero (ZEEP). After 5 min in each inspiratory pressure step, a 4 s inspiratory pause was performed to obtain a thoracic juxta-diaphragmatic single slice helical CT image and to collect respiratory mechanics data and an arterial blood sample. Lung parenchyma aeration was defined as overinflated, normally-aerated, poorly-aerated, and non-aerated according to the CT attenuation number (−1,000 to −900 HU, −900 to −500 HU, −500 to −100 HU, and −100 to +100 HU, respectively).

**Result:**

At 5 cmH_2_O airway pressure, tidal volume was 6.7± 2.2 ml kg^−1^, 2.1% (0.3–6.3%) of the pulmonary parenchyma was overinflated and 84.9% (77.6%−87.6%) was normally inflated. Increases in airway pressure were associated with progressive distention of the lung parenchyma. At 15 cmH_2_O airway pressure, tidal volume increased to 31.5± 9.9 ml kg^−1^ (*p* < 0.001), overinflated pulmonary parenchyma increased to 28.4% (21.2–30.6%) (*p* < 0.001), while normally inflated parenchyma decreased 57.9% (53.4–62.8%) (*p* < 0.001). Tidal volume and overinflated lung fraction returned to baseline when airway pressure was decreased. A progressive decrease was observed in arterial carbon dioxide partial pressure (PaCO_2_) and end-tidal carbon dioxide (ETCO_2_) when the airway pressures were increased above 9 cmH_2_O (*p* < 0.001). The increase in airway pressure promoted an elevation in pH (*p* < 0.001).

**Conclusions and Clinical Relevance:**

Ventilation with 5 and 7 cmH_2_O of airway pressure prevents overinflation in healthy cats with highly compliant chest walls, despite presenting acidemia by respiratory acidosis. This fact can be controlled by increasing or decreasing respiratory rate and inspiratory time.

## Introduction

Mechanical ventilation (MV) aids in maintaining adequate pulmonary gas exchange during general anesthesia for surgical procedures. However, it may lead to the development of areas of atelectasis and hyperinflation within the pulmonary parenchyma (1–4). Hyperinflation can lead to pulmonary lesions by cyclical stretching of lung tissues, promoting inflammation, acute lung injury, and postoperative complications (5–7). Despite the evidence in other species, to the authors' knowledge, there is no evidence describing the pulmonary aeration distribution of healthy cats undergoing positive pressure ventilation in relation to lung hyperinflation and alveolar collapse.

The current ventilation monitoring methods available at the bedside are unable to detect lung hyperinflation. The quantitative assessment of the distribution of aeration within the lungs during MV can be evaluated using CT (8). Using this technique, it is possible to precisely compute the amount of lung parenchyma overinflation in diverse inspiratory pressure conditions, allowing the identification of less harmful ventilatory strategies (9–12). The aim of this study was to assess the inspiratory lung aeration distribution by helical CT and respiratory mechanics in anesthetized cats ventilated with 6 different levels of inspiratory pressure (5, 7, 9, 11, 13, and 15 cmH2O) to obtain the best value of inspiratory pressure to ventilate healthy lungs. The hypothesis of the study was that low inspiratory pressures would result in more areas of lung collapse, while high inspiratory pressures would result in more areas of overinflation in healthy cat lungs.

## Materials and Methods

The study was approved by the Ethics Committees for Animal Research at the Faculty of Veterinary Medicine and Animal Science of the University of São Paulo (FMVZ—USP protocol number 110/8) and Faculty of Medicine of the University of São Paulo (CEUA—USP protocol number 100/10). Informed consent was obtained for the enrolled cats. It was conducted at the Radiology Service of the Department of Surgery—Faculty of Veterinary Medicine and Animal Science of the University of São Paulo, São Paulo, Brazil.

### Animals

A total of 25 intact male cats scheduled for orchiectomy in the University hospital, aging 1–5 years were investigated in this study. The inclusion criteria were the absence of respiratory and cardiovascular disease history. The presence of abnormalities observed in tomographic scouts of the thorax, in baseline arterial blood gases, or preoperative blood cell count, renal and hepatic plasma chemistry panel were considered as the exclusion criteria.

### Anesthetic Protocol

One day before the experiment, the animals underwent catheterization of the cephalic vein and dorsal pedal artery using a 22-gauge catheter (BD Insyte® Autoguard, Juiz de Fora, MG, Brazil) under sedation with dexmedetomidine hydrochloride (Dexmedetor® Orion Pharma, Espoo, Finland), (10 μg kg^−1^) intramuscularly (IM). Sedation was reversed with atipamezole (Antisedan® Orion Pharma, Espoo, Finland) (10 μg kg^−1^) IM and then the animals were placed in cages with food and water ad libitum. The animals fasted for 8 h before the experiments.

On the day of the experiment, the animals were anesthetized with propofol (5 mg kg^−1^) intravenously (IV) (Propovan; Cristália; São Paulo, Brazil) and underwent endotracheal intubation with a 3.5 mm size, 19 cm length, and a cuffed cannula (Solidor, Bonree Medical, Guangdong, China). Anesthesia was maintained with a continuous infusion of propofol (0.5 mg kg^−1^ min^−1^). Paralysis was promoted by the intravenous administration of 1 mg kg^−1^ of rocuronium (Rocuron; Cristália, São Paulo, Brazil) and supplemented with increments of 0.5 mg kg^−1^ if the animal presented any signs of spontaneous breathing effort based on the airflow waveform.

After the CT scan protocol, animals were transported to the operation room where neutering surgery was performed.

### Study Protocol

Prior to the beginning of each study, standard tests of the ventilator (Galileo®, Hamilton Medical, Bonaduz, Switzerland) were performed (pneumotachograph calibration, leak testing, and calculation of the breathing circuit compliance). After the initial ventilator tests, a calibrated Wright spirometer (nSpire, Hertford, UK) was connected between the pneumotachograph and the breathing circuit (neonatal, 150 cm length) to verify the accuracy of V_T_ measurement computed by the ventilator. A variation of up to 5% between the V_T_ values expressed by the ventilator and the Wright spirometer was accepted.

### Experimental Design

After anesthetic induction and positioning of the patient in the dorsal recumbency, pressure-control ventilation (PCV) was initiated with an inspiratory pressure of 5 cmH_2_O, zero end-expiratory pressure (ZEEP) f_R_ of 15 breaths per min, and an inspiratory time of 1 s and FiO_2_ 40%.

**Figure 1:**
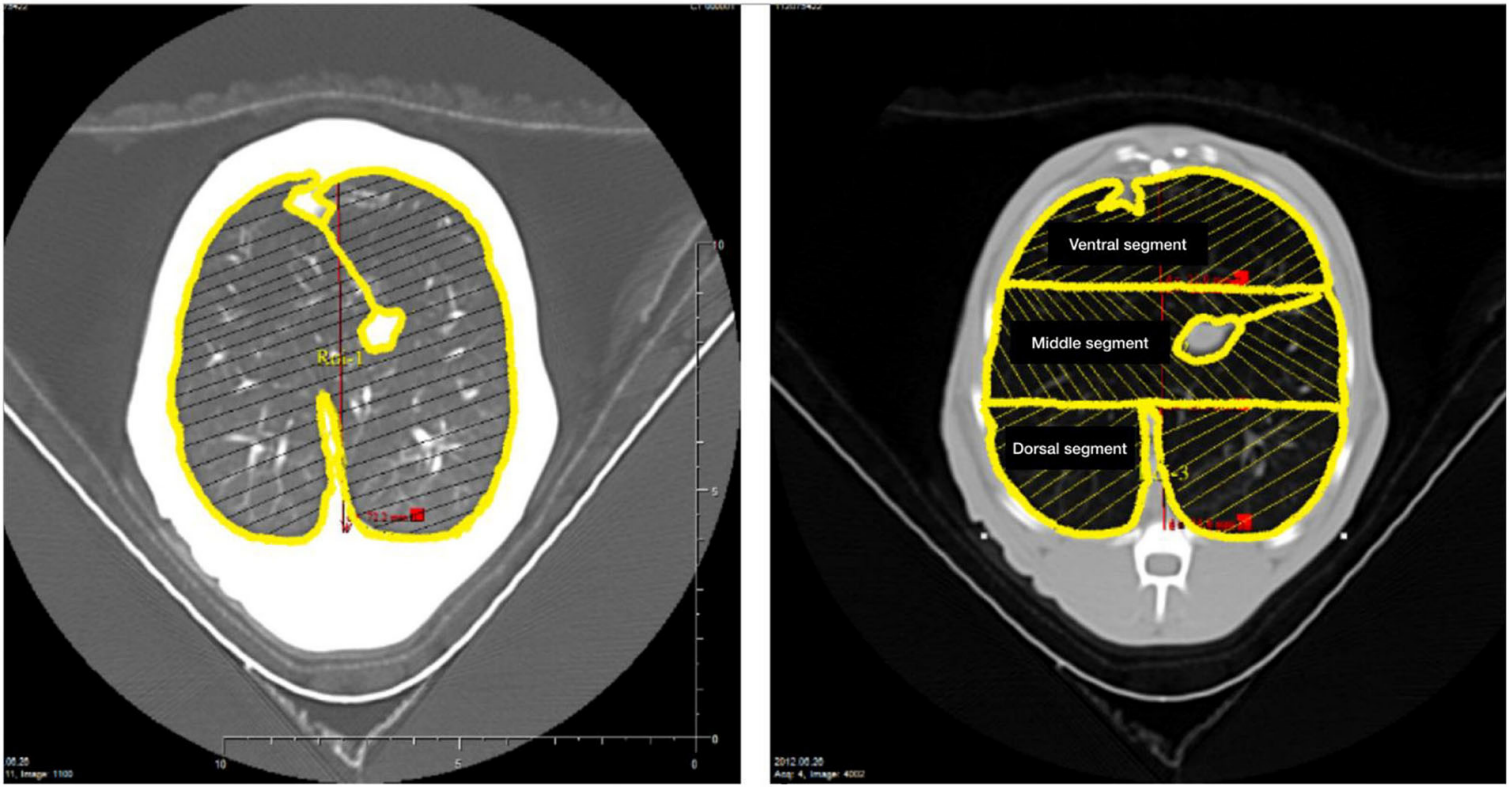
The left panel shows the delineation of the region of interest encompassing the left and right lungs performed in a juxta-diaphragmatic CT section. The right panel shows the delineation of 3 regions of interest of equal height distributed along the ventral-dorsal axis (ventral, middle, and dorsal lung segments) encompassing the left and right lungs.

After 20 min of anesthetic induction and hemodynamic stabilization, inspiratory pressure was progressively increased by increments of 2 cmH_2_O every 5 min until reaching 15 cmH_2_O. After that, inspiratory pressure was reduced in a descending stepwise manner in steps of 2 cmH_2_O until 5 cmH_2_O. At the end of each period, an arterial blood sample was collected using 1 ml syringes (Becton Dickinson, Curitiba, Brazil), washed with heparin for blood gas analysis, and an inspiratory pause of 4 s was performed to obtain a thoracic CT image (Figure 1).

At each inspiratory pressure level, the arterial pressure (AP), heart rate (HR), and pulse oximetry (SpO_2_) of the animals were evaluated using a multiparameter monitor (Model 2020, Dixtal, Manaus, Brazil). The blood pressure transducer (TruWave®, Edwards, California, USA) was connected to the arterial catheter by noncompliant fluid-filled tubing and was zeroed and positioned at the heart level (elbow joint).

The inspired oxygen fraction (FiO_2_, %) oxygen and end-tidal partial pressure of carbon dioxide (EtCO_2_, mmHg) were monitored using a side stream gas analyzer (Dixtal, Manaus, Brazil).

The peak and plateau pressure (Pplat, cmH_2_O), minute volume, expiratory tidal volume, and respiratory rate were obtained directly from the ventilator. The static compliance was calculated using the following formula:


(1) $$\begin{array}{r} {\text{C}}_{\text{stat}}={\text{V}}_{\text{E}}/\left({\text{P}}_{\text{plat}}-\text{PEEP}\right),{\text{or simplifying, C}}_{\text{stat}}={\text{V}}_{\text{E}}/P_{\text{plat}} \end{array}$$

where C_stat_ is the static compliance, V_E_ is the expiratory volume, P_plat_ is the plateau pressure obtained at the end of a 4 s-inspiratory pause with zero flow, and PEEP is the positive end-expiratory pressure, which was zero through the study.

Arterial blood samples (0.7 mL) were anaerobically collected in heparinized syringes, and blood gas analyses of the pH, partial pressure of oxygen (PaO_2_), partial pressure carbon dioxide (PaCO_2_), bicarbonate concentration (HCO${}_{3}^{-}$), standard base excess (SBE), and oxygen saturation (SaO_2_) were immediately performed (ABL5, Radiometer, Denmark).

### CT Image Acquisition and Analysis

After intubation, the cats were placed in dorsal recumbency throughout the entire procedure with their thoracic limbs extended forward carefully to ensure that the spine and head of each animal were in a straight line to acquire symmetrical images. The thoracic CT scan was obtained in dorsal recumbency because most surgeries (like laparotomies) are routinely done in this recumbency. The CT images were obtained at the end of a 4-s inspiratory pause at each inspiratory pressure level, from 1 cm cranial the diaphragmatic dome using a single slice helical CT scanner (Xpress/GX, Toshiba, Japan) with the same settings (120kVp and 150 mA, matrix size 512 × 512). Images were reconstructed at 5 mm thickness and kernel FC50 for standard lung imaging. The images were analyzed using the software Osirix (Osiris 4.19, University Hospital of Geneva, Switzerland). Briefly, each pulmonary region of interest (ROI) was manually delineated and the pixels contained in it were distributed into 1,200 compartments according to their X-ray attenuation coefficient (CT number). Each pixel is characterized by a CT number that represents the attenuation coefficient of the X-ray by the structure being studied minus the attenuation coefficient of water, which is 1, divided by the attenuation coefficient of water expressed in Hounsfield units (HU) (13). Therefore, for each compartment of a known CT number, it was possible to compute tissue and gas volumes and tissue mass using the following formulas:

(1) Volume of the voxel = (size of the pixel)^2^ x section thickness, with an area provided for each pixel tomographic study
(2) Total volume of the compartment = number of voxels × voxel volume for each track radiological density
(3) Volume of gas = (CT coefficient/1,000) × total volume of the compartment, if the compartment has a considered CT coefficient between 0 and −1,000 HU; or Volume of gas = 0, if the compartment in question has a CT coefficient >0 HU; or volume of gas = total volume of the compartment if the CT coefficient is −1,000 HU
(4) Volume of tissue = (1 + CT coefficient / 1,000) × total volume of the compartment, if the compartment considered has a CT coefficient between 0 and −1,000 HU; or Volume of tissue = number of voxels × voxel volume, if compartment is considered to have a CT coefficient of >0 HU; or volume of tissue = 0 if the compartment has a CT coefficient less of than −1,000 HU
(5) Weight of tissue = volume of tissue, if the compartment considered has CT coefficient <0 HU; or weight of tissue = (1 + CT coefficient / 1,000) × total volume of the compartment, if the compartment in question has a CT coefficient >0 HU.

The total lung tissue and volumes of each given ROI were computed by adding all the partial masses and volumes of the compartments. Lung parenchyma was further analyzed according to its aeration, defined as follows: 1) overinflated parenchyma characterized by CT numbers between −1000 HU and −900 HU (9, 14, 15), 2) normally-aerated parenchyma characterized by CT numbers between −900 HU and −500 HU, 3) poorly-aerated parenchyma characterized by CT numbers between −500 HU and −100 HU (16) and 4) non-aerated parenchyma characterized by CT numbers between −100 and + 100 HU (17).

In the initial analysis, a large ROI encompassing both the left and right lungs was delineated in the CT images obtained in each peak inspiratory pressure level, to evaluate the overall lung volumes and tissue mass, as shown in Figure 1. The portions of the pulmonary hila containing the trachea, main bronchi, and hilar blood vessels were excluded from the ROI. In a second analysis, the left and right lungs were segmented in 3 regions of interest of equal height distributed along the ventral-dorsal axis (ventral, middle, and dorsal, relative to patient's anatomy) and the overinflated lung tissue mass fraction were computed in each airway pressure condition (Figure 1).

### Statistical Analysis

The normal distribution of data was evaluated by means of visual analysis and the Shapiro-Wilks test. All data were expressed as the mean value ± SD or as median (25–75% interquartile), according to their distribution. The physiological variables, blood gases variables, and lung CT-derived variables were compared at different levels of inspiratory pressure by means of one-way ANOVA or Friedmann test, followed by multiple comparisons when indicated.

The fraction of the overinflated parenchyma to the overall parenchyma mass within the 3 lung segments (ventral, middle, and dorsal) obtained at each peak inspiratory pressure level was compared using the Kruskal-Wallis test followed by Dun's test. A *p*-value of < 0.05 was considered significant. All statistical analyses were performed using IBM SPSS package version 22 (IBM Corp, Armonk, NY, USA) and GraphPad Prism 6 for Mac (GraphPad Software, La Jolla, California, USA).

## Results

From the 25 cats included in this study, only the data from 17 cats were analyzed. Eight cats had to be excluded due to image artifacts (5 cats), pulmonary infiltrates detected on the baseline CT images (2 cats), and hypotension refractory to fluid therapy and ephedrine (1 cat). The animals had an average age of 3.2 ± 1.3 years and a weight of 3.5 ± 0.5 kg.

### Lung Aeration Distribution

The increase in the inspiratory pressure caused an elevation in the total CT section volume, being 34.2 % greater than baseline at the airway pressure of 15 cmH_2_O. The decrease in airway pressure resulted in the return of the lung volume to baseline (*p* < 0.001). This variation in the total volume was due to an increase in the volume of gas, while the volume of tissue decreased but to a minor degree (Table 1).

**Table 1:** Lung tissue and gas volumes, tissue mass and lung analysis according to aeration at different airway pressures.

	**Inspiratory pressure**	**5** **cmH_**2**_O**	**7** **cmH_**2**_O**	**9** **cmH_**2**_O**	**11** **cmH_**2**_O**	**13** **cmH_**2**_O**	**15** **cmH_**2**_O**	**13** **cmH_**2**_O**	**11** **cmH_**2**_O**	**9** **cmH_**2**_O**	**7** **cmH_**2**_O**	**5** **cmH_**2**_O**	***p*-value**
Volumetric CT analysis	Total volume (mL)	14 ± 2.8	15.3 ± 2.9^*^	16 ± 3.1^*^	16.9 ± 3.1^*^	17.5 ± 3.1^*^	18.8 ± 3.6^*^	17.3 ± 3^*^	18 ± 3.5^*^	16.8 ± 3.2^*^	16.5 ± 3.1^*^	15.1 ± 3.1	*p <* 0.001
	Tissue volume (mL)	3.1 ± 0.7	2.9 ± 0.7	2.8 ± 0.6	2.5 ± 0.6^*^	2.5 ± 0.8^*^	2.3 ± 0.6^*^	2.3 ± 0.5^*^	2.7 ± 0.7	2.8 ± 0.7	2.9 ± 0.6	3.1 ± 0.6	*p <* 0.001
	Gas volume (mL)	10.9 ± 2.6	12.4 ± 2.9^*^	13.3 ± 3.3^*^	14.3 ± 3.1^*^	15 ± 3.2^*^	16.6 ± 3.4^*^	15.7 ± 3.4^*^	14.5 ± 3^*^	14 ± 3.3^*^	13.6 ± 3^*^	12 ± 3	*p <* 0.001
	Total tissue (g)	3.1 ± 0.7	2.9 ± 0.7	2.8 ± 0.6	2.5 ± 0.6^*^	2.5 ± 0.8^*^	2.3 ± 0.6^*^	2.3 ± 0.5^*^	2.7 ± 0.7	2.8 ± 0.7	2.9 ± 0.6	3.1 ± 0.6	*p <* 0.001
Lung analysis according to CT attenuation	OIP (%)	2.1 (0.3–6.3)	6.3 (1.7–15.3)^*^	9.6 (4.8–16.1)^*^	14.9 (6.2–24.9)^*^	16.1 (6.2–22.5)^*^	28.4 (21.2–30.6)^*^	22.5 (16.8–25.6)^*^	11 (3.4–21.4)^*^	9.3 (3.6–17.7)^*^	8.6 (3.6–11.3)	5.1 (2.3–9.3)	*p <* 0.001
	NoIP (%)	84.9 (77.6–87.6)	79.9 (72.2–85.6)	77.3 (66.9–82.6)	74.1 (56.7–78.1)^*^	70.1 (56.1–79.8)^*^	57.9 (53.4–62.8)^*^	64.1 (58.4–68.1)^*^	78 (68.8–83.7)	74 (64.9–85.2)	80 (70.9–85)	81 (76.2–87.4)	*p <* 0.001
	PIP (%)	10 (7.8–11.5)	10 (9.0–11.9)	10 (8.8–12.6)	11 (8.5–13)	11 (10–14.1)	11 (10.8–12.7)	11 (10.3–12.2)	9.5 (8.8–10.9)	10 (9.2–10.9)	9.4 (8.2–11.4)	9 (8.2–11.4)	*p* = 0.08
	NIP (%)	2.3 (0.9–3.9)	2.4 (1.8–5)	2.6 (0.7–4)	3.3 (2–7.5)	2.6 (1.7–3.6)	4.5 (2.6–8.8)	3 (1.7–4.7)	1.9 (0.8–2.7)	2.9 (1.9–6.9)	2.6 (1.3–4.4)	2 (1–5.6)	*p* = 0.25

*OIP, overinflated parenchyma; NoIP, normally-inflated parenchyma; PIP, poorly-inflated parenchyma; NIP, non-inflated parenchyma*.

* *Different from baseline airway pressure with p < 0.05*.

The overinflated tissue fraction increased from 2.1% at the airway pressure of 5 cmH_2_O to 28.4% at the airway pressure of 15 cmH_2_O (*p* < 0.001), with a reduction of normally-aerated parenchyma from 84.9 to 57.9%. The reduction of airway pressure decreased the amount of overinflated lung parenchyma and restored the normally-aerated parenchyma fraction to baseline values at 5 cmH_2_O airway pressure. The fraction of non-aerated and poorly-aerated parenchyma did not change significantly during the study (Table 1).

### Regional Overinflation in the Ventral, Middle and Dorsal Lung Regions

**Figure 2:**
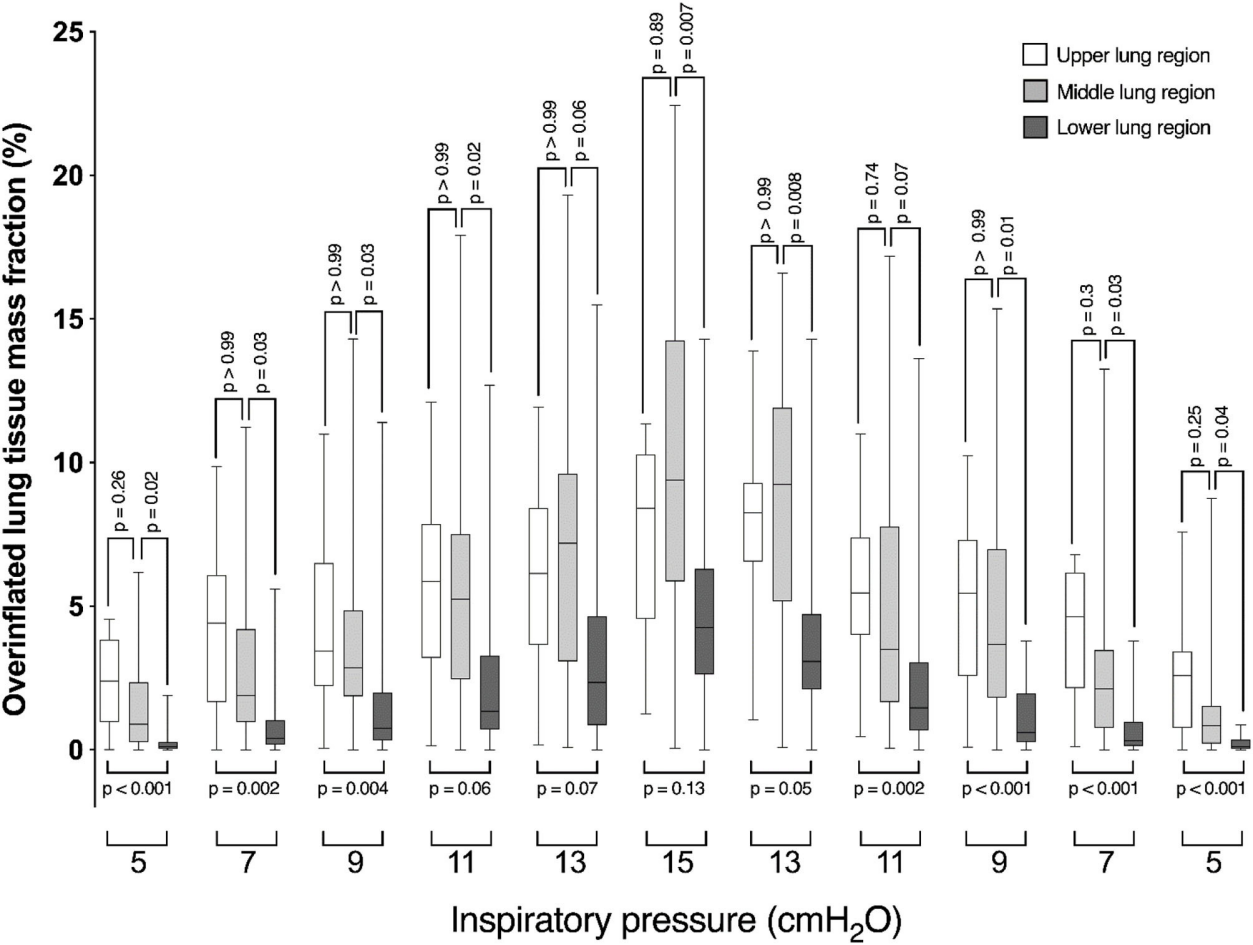
Box-plot graph showing the fraction of over-inflated lung tissue mass of ventral (open box-plots), middle (light gray box-plots), and dorsal (dark gray box-plots) lung segments in diverse airway pressure conditions.

The amount of overinflated parenchyma progressively increased within the ventral, middle, and dorsal lung segments in proportion to the increase in airway pressure up to 11 cmH_2_O in a heterogeneous fashion, as shown in Figure 2. From 5 cmH_2_O up to 9 cmH_2_O airway pressure, the proportion of overinflated parenchyma was greater in the ventral and middle lung regions. From 11 cmH_2_0 airway pressure up to 15 cmH_2_O, the overinflation occurring in the dorsal lung region reached the same proportion as observed in the ventral region. At 13 cmH_2_O airway pressure, the proportion of overinflated parenchyma observed in the middle lung region was greater than that in the ventral region. A stepwise decrease in inspiratory pressure toward baseline reversed the alterations in the distribution of regional overinflation within the lung parenchyma.

### Respiratory Mechanics

The plateau pressure increased with the elevation of inspiratory airway pressures and returned to baseline after the stepwise decrease in inflation pressure *(p* < 0.001) (Table 1). The increase in airway pressure promoted a significant increase in tidal volume, from 6.7 ± 2.2 mL/kg at 5 cmH_2_O airway pressure to 31.5 ± 9.9 mL/kg at 15 cmH_2_O airway pressure (*p* < 0.001), returning to baseline values at the pressure of 5 cmH_2_O (Table 2).

**Table 2:** Hemodynamic, respiratory, and laboratorial variables evaluated at different airway pressures.

**Inspiratory pressure**	**5** **cmH_**2**_O**	**7** **cmH_**2**_O**	**9** **cmH_**2**_O**	**11** **cmH_**2**_O**	**13** **cmH_**2**_O**	**15** **cmH_**2**_O**	**13** **cmH_**2**_O**	**11** **cmH_**2**_O**	**9** **cmH_**2**_O**	**7** **cmH_**2**_O**	**5** **cmH_**2**_O**	***P*-value**
Heart rate (bpm)	149 ± 23	150 ± 19	156 ± 11^*^	159 ± 9^*^	162 ± 11^*^	164 ± 12^*^	162 ± 10^*^	160 ± 10^*^	159 ± 10^*^	157 ± 9^*^	151 ± 14	*p* < 0.001
MAP (mmHg)	78 ± 11	77 ± 10	77 ± 11	79 ± 19	83 ± 24	80 ± 19	81 ± 17	82 ± 17	84 ± 18	86 ± 20	88 ± 21	*p* = 0.55
Tidal Volume/weight (mL/kg)	6.7 ± 2.2	10.9 ± 3.9	16.9 ± 4.6^*^	21.6 ± 6^*^	27 ± 8^*^	31.5 ± 9.9^*^	27.9 ± 9.1^*^	22.7 ± 7.6^*^	18.5 ± 6.1^*^	13 ± 4.4^*^	8.2 ± 2.9	*p* < 0.001
Plateau pressure (cmH_2_O)	5 ± 1	7 ± 1^*^	9 ± 1^*^	11 ± 1^*^	13 ± 1^*^	14 ± 1^*^	12 ± 1^*^	10 ± 1^*^	8 ± 1^*^	7 ± 1^*^	5 ± 1	*p* < 0.001
Static compliance (mL/cmH_2_O)	5.2 ± 6.1	5.9 ± 1.7	7.3 ± 1.7	7.7 ± 2^*^	8.3 ± 2.5^*^	8.5 ± 2.7^*^	8.8 ± 2.7^*^	8.4 ± 2.7^*^	8.3 ± 2.6^*^	7.4 ± 2.8	6.3 ± 2.1	*p* < 0.001
Risnp (cmH_2_O/L/sec)	40 ± 20.5	40 ± 18.8	47 ± 24.5	48 ± 25.8	53 ± 35.2^*^	54 ± 37.2^*^	50 ± 30.6^*^	44 ± 21.5	43 ± 20.1	40 ± 21.6	39 ± 23.6	*p* < 0.001
pH	7.24 ± 0.07	7.28 ± 0.07^*^	7.3 ± 0.07^*^	7.40 ± 0.07^*^	7.45 ± 0.07^*^	7.49 ± 0.06^*^	7.48 ± 0.07^*^	7.47 ± 0.06^*^	7.43 ± 0.07^*^	7.41 ± 0.05^*^	7.35 ± 0.05^*^	*p* < 0.001
PaCO_2_ (mmHg)	48 ± 10	42 ± 10^*^	36 ± 9^*^	31 ± 8^*^	26 ± 7^*^	23 ± 7^*^	23 ± 6^*^	24 ± 6^*^	26 ± 8^*^	28 ± 7^*^	34 ± 8^*^	*p* < 0.001
ETCO_2_ (mmHg)	42 ± 12	37 ± 12^*^	29 ± 10^*^	25 ± 9^*^	22 ± 8^*^	19 ± 7^*^	19 ± 7^*^	19 ± 7^*^	21 ± 8^*^	28 ± 8^*^	27 ± 9^*^	*p* < 0.001
PaO_2_ (mmHg)	179 ± 36	202 ± 30^*^	209 ± 27^*^	219 ± 29^*^	219 ± 24^*^	227 ± 21^*^	223 ± 27^*^	219 ± 24^*^	223 ± 23^*^	217 ± 28^*^	200 ± 33	*p* < 0.001
PaO_2_/FiO_2_ ratio	444 ± 87	506 ± 79^*^	523 ± 73^*^	545 ± 71^*^	546 ± 61^*^	571 ± 54^*^	553 ± 70^*^	548 ± 62^*^	560 ± 58^*^	547 ± 66^*^	496 ± 88	*p* < 0.001
SBE (mmol/L)	−7.4 ± 1.4	−7.4 ± 1.5	−6.9 ± 1.53	−6.9 ± 1.52	−6.8 ± 1.83	−7 ± 1.82	−6.7 ± 1.63	−6.7 ± 1.84	−7.1 ± 1.45	−6.8 ± 1.44	−6.8 ± 1.64	*p* = 0.91
HCO^3^ (mmol/L)	18.9 ± 1.5	18.5 ± 1.8	18.2 ± 1.9	17.4 ± 1.8	16.8 ± 1.89^*^	16.4 ± 2^*^	16.6 ± 2^*^	16.7 ± 2.1^*^	16.6 ± 1.6^*^	17.1 ± 1.7^*^	18 ± 1.6	*p* < 0.001

*MAP, mean artery pressure; Rinsp - inspiratory resistance; PaCO_2_, arterial carbon dioxide partial pressure; ETCO_2_, end-tidal carbon dioxide concentration; PaO_2_, arterial oxygen partial pressure; PaO_2_/FiO_2_ ratio; SBE, standard base excess; HCO^3^, arterial bicarbonate*.

* *Different from baseline airway pressure with p < 0.05*.

The static compliance increased from 11 cmH_2_O airway pressure until 15 cmH_2_O of airway pressure. After that pressure level, the static compliance remained higher than baseline until 9 cmH_2_0 (*p* < 0.001). When the airway pressure decreased below 7 cmH_2_O, the static compliance decreased returning to baseline values at 5 cmH_2_O (Table 2).

### Cardiovascular Variables and Blood Gases

Hemodynamic variables of the animals during the study are shown in Table 2. No changes were observed in mean arterial pressure in different airway pressure conditions, however, heart rate increased as peak airway pressure increased from 9 cmH_2_0 airway pressure up to 15 cmH_2_0 and returned to baseline as airway pressure was decreased to 5 cmH_2_O (*p* < 0.05).

A progressive decrease was observed in PaCO_2_ and ETCO_2_ when the airway pressures were increased above 9 cmH_2_O (*p* < 0.001). The increase in airway pressure promoted an elevation in pH (*p* < 0.001). During the stepwise airway pressure decrease, PaCO_2_ and ETCO_2_ increased and pH values decreased but did not return to baseline. An increase in arterial PaO_2_ was observed when airway pressure was raised until 9 cmH_2_O (*p* < 0.001), remaining stable until the end of the experiment. No variations were observed in base excess throughout the experiment, while HCO^3^ decreased with airway pressure above 11 cmH_2_O, returning to baseline level at 5 cmH_2_O (Table 2).

## Discussion

In this study, we observed in anesthetized and mechanically ventilated cats with increasing airway pressures: (1) a linear increase of tidal volume; (2) a linear increase in overinflation of the lung parenchyma; (3) a reduction of normally aerated areas; (4) upholding of the poorly and non-aerated areas. The airway pressure values of 5 and 7 cmH_2_O generated the most normally aerated lung areas (84.9 and 79.9 %) and tidal volumes (6.7 and 10 ml/kg respectively). To the best of the author's knowledge, there are no similar studies in cats.

For several decades, it has been recognized that MV plays an important role in the development of lung injury in artificially ventilated individuals (18). In 1974, Webb and Tierney described the impact of high pressures and volumes in the lungs of healthy rats ventilated with inspiratory pressures of 14, 30, or 45 cmH_2_O with and without PEEP (19). They observed that animals ventilated with high inspiratory pressure developed alveolar and perivascular edema, severe hypoxemia, and decreased compliance, while those ventilated with low pressure showed no pathologic lung changes. In subsequent years, it was observed in several other investigations that the use of high tidal volumes was associated with alterations of vascular permeability and with the development of interstitial edema (20–22). Data from recent studies have shown that the harm caused by high tidal ventilation goes beyond alveolar and endothelial cell damage, being implicated in mitochondrial injury with mitochondrial DNA release (23) and in remodeling of the lung extracellular matrix (24). Much of these findings were attributed to the cyclical tidal overstretch of the parenchyma during the respiratory cycle. Despite the evidence showing the injurious potential, MV remains a cornerstone in anesthesia and critical care medicine.

To reduce postoperative pulmonary complications such as pneumonia and postoperative pulmonary hypoxemia, the use of reduced tidal volumes ranging from 6 to 8 ml/kg has become a current practice in human anesthesia (7, 25, 26). Comparative respiratory physiology studies across different mammal species also indicate that spontaneous breath tidal volumes range from 6 to 8 ml/kg (27, 28). Even though, in small animals undergoing general anesthesia, the recommended tidal volumes to be used are in the range of 10 to 15 ml/kg (29, 30). It is unknown what the effect of such high tidal volumes and airway pressures is on the pulmonary structure and its possible long-term consequences in healthy cats undergoing general anesthesia and MV for surgical procedures, and studies like this one are important to determine the ideal inspiratory pressure and V_T_ to be clinically used in this species. We showed here that in healthy cats ventilated with PCV, inspiratory pressure of 5–7 cmH_2_O can be safely used, generating tidal volume between 6–10 ml/kg. However, an individual approach to determine the best ventilatory settings should be used for each patient, monitoring airway pressures and tidal volumes.

Quantitative CT has been used clinically and experimentally to investigate the distribution of the ventilation within the lung parenchyma. However, there are no clear definitions on the radiological limits of pulmonary parenchyma hyperinflation in small animals, and one could argue that considering the distribution of aeration within the lungs of healthy cats, it behaves in the same fashion as in humans and could be a limitation of this study. Nevertheless, other authors have described the same CT attenuation thresholds to define lung aeration compartments and as well as the relative volume in these pulmonary compartments according to aeration in cats (31, 32).

In this study, we observed that the relative distribution of pulmonary aeration was similar to that observed in humans, being the lung composed of 85% normally-aerated parenchyma, 10% of poorly-aerated parenchyma, about 2% of non-aerated, and 2% of overinflated parenchyma (9). These 2 last compartments are mainly composed of blood within pulmonary vessels and air filling the large airways, respectively. An increase in airway pressure from 5 to 15 cmH_2_0 increased the tidal volume from 6.7 to 31.5 ml/kg. As a result of the progressive pressurization of the airways, there was a massive increase in the amount of overinflated parenchyma and an expressive reduction of the normally-aerated parenchyma. Probably, as airway pressure increased, a portion of the normally-aerated lung parenchyma became overinflated, without any significant changes in the poorly-aerated parenchyma or in the non-aerated parenchyma. In this way, we can assume that when the airway pressure was increased, the amount of pulmonary parenchyma exposed to tidal overinflation increased correspondingly as well as the risk of ventilator-induced pulmonary injury.

Furthermore, increases in airway pressure promote differential patterns of inflation in the dorsal, middle, and ventral lung regions. From 5 to 11 cmH_2_O, overinflation occurs preferentially in the ventral lung segment, the most compliant pulmonary segment physiologically. At this point, the ventral segment is fully distended and overinflation reaches a plateau and additional increases in airway pressure do not increase the fraction of overinflated lung. Meanwhile, the middle lung segment keeps on over-distending up to 15 mmHg airway pressure. At the pressure of 13 cmH_2_O onwards, dorsal lung segment overinflation is comparable to that of the ventral segment, indicating that the lungs reached entirely overinflated. Even though, ventilatory driving pressures in these ranges are currently being used in veterinary clinical practice (29).

Another phenomenon that must be argued is that some part of the normally-aerated parenchyma decreased as airway pressure increased due to de-recruitment caused by the enlarging overinflated lung segment, resulting in increases in poorly- and non-aerated parenchyma fractions. According to our data, poorly- and non-aerated lung fractions remained largely unaltered as airway pressure variated, rejecting a possible de-recruitment of normally-aerated lung parenchyma in the studied airway pressure variation range. In the present study, no significant areas of alveolar collapse were observed, whereas the largest area of collapse represented an average of 2.38 ± 3.6% when the inspiratory pressure reached 15 cmH_2_O. We did not use PEEP, even a fixed low PEEP, as the objective of the study was not to evaluate lung atelectasis. This study was designed to evaluate hyperinflation resulting from increasing airway pressures during pressure-controlled ventilation. With 15 cmH2O inspiratory pressure, the tidal volume was 31.5 ± 9.9 ml. Current practices for safe driving pressure (plateau pressure minus PEEP) range from 14 to 18 cmH_2_O in acute respiratory distress syndrome (ARDS) and healthy human patients (33, 34). Higher inspiratory pressure would only be harmful to the cats in our study without additional recruitment benefit.

It is believed that the use of a FiO_2_ of 40%, the short duration of anesthesia (75 min), the low weight of the abdominal organs of cats, and their high thoracic and lung compliance, prevented alveolar collapse at any time during the study. Even though the most prevalent abnormality detected in thoracic CT scans of healthy anesthetized cats was pulmonary atelectasis (41%) (35), different studies have shown significant increases in collapsed lung areas during ventilation with FiO_2_ values of 100% (7.1 ± 2.7%) under spontaneous ventilation in felines (36) and in mechanically controlled ventilation (12.8 ± 3.7%) in dogs with a tidal volume of 15 mL/kg (37). In both studies, the tendency of collapsed alveoli was the same at 100% FiO_2_, although the most striking values were observed in dogs. The most likely reason for this result is associated with the increased visceral weight in dogs compared with cats, resulting in a greater compressive effect on the lung lobe bases.

In terms of the hemodynamic effects caused by the MV, the use of high pressures and volumes is related to hypotension caused by low output. Pulmonary hyperinflation can cause increased pulmonary vascular resistance and increased pulmonary artery pressure, impairing the right ventricular ejection (38). However, this result was not observed in the present study given the short time during which the animals were exposed to each time pressure, besides the fact that all of the patients being in normovolemic status and HR has increased by 10% at the highest airway pressure (13–15 cmH_2_O) in a compensatory way.

The static compliance of the respiratory system increased with the elevation of inspiratory airway pressure at 63% at the highest pressure of 15 cmH_2_O when compared to the baseline. The referred increase is in agreement with the observed ones also in the tidal volume and overall CT section volume. These factors also accompanied the increase in overinflated areas showing that 15 cmH_2_O inspiratory pressure could be harmful to the healthy lungs of cats. A comparison with the values of compliance of the respiratory system in the veterinary literature is difficult because of the different methods used, although their values range from 5.1 to 6.8 ml/cmH_2_O (39).

In cats, a PaCO_2_ below 25 mmHg and pH above 7.46 are the limits for respiratory alkalosis, and beyond those values (PaCO_2_ <20–25 mmHg), arteriolar vasoconstriction may occur, reducing cerebral and myocardial blood flow (40). In this study, we observed a progressive decrease in PaCO_2_ when the airway pressures were increased, reaching values close to life-threatening levels at 15 cm H_2_O airway pressure.

Based on the arterial PaCO_2_ analysis, the best airway pressure was 9 and 11 cmH_2_O stepwise increased, but in this level, there were 9.6–14.9% of overinflated parenchyma compared to 5 cmH_2_O airway pressure (2.1%) and less normally-inflated parenchyma. However, in the best aeration of the pulmonary parenchyma at 5 and 7 cmH_2_O airway pressure, there was respiratory acidosis. Respiratory acidosis, or primary hypercapnia, occurs when carbon dioxide production exceeds elimination via the lung and is mainly owing to alveolar hypoventilation (41). Despite this, respiratory acidosis can be reversed by increased alveolar minute ventilation, as increasing respiratory rate as the product of and the portion of tidal volume that reaches perfused gas exchange units (42).

A significant increase in oxygenation during the airway stepwise increase was observed and was maintained during the decreases in pressure. Although oxygenation was normal at the baseline this increase probably occurred most likely because of the recruitment of collapsed areas not evaluated by the single CT images of the studied region.

### Limitations

This study has some limitations: (A) Tomographic analyses were performed on a single CT image close to the diaphragm. Despite the CT scanning of the whole thorax enabling the evaluation of the distribution of ventilation throughout the entire lungs (43), the time to acquire the image in elevated airway pressure conditions may cause unnecessary hemodynamic derangements. Secondly, it would expose the cats to almost 10x higher doses of radiation and to an avoidable higher risk of cancer (44). Therefore, we decided to obtain fewer images to lower animal exposure to X-rays; (B) The CT section thickness used in this study was 5 mm. This spatial resolution might result in a significant underestimation of the actual overinflation occurring within the lungs when compared to higher spatial resolution with a 2 mm section (45). Conversely, the use of a thicker CT section may minimize the bias introduced in the CT analysis by the cephalic-caudal displacement of the lung parenchyma to the outside of limits of a thinner CT section; (C) Another problem related to the analysis of a single CT section is the partial volume effect that can interfere with the computation of the CT number in voxels nearing the boundaries of the ROI. These voxels may be composed of gas and much more dense structures such as bones. Therefore, it can underestimate the actual CT number values. Since the cross-sectional area of the feline thorax is much smaller than in humans, this effect may be potentially greater. The relative values calculated in one CT section may not be representative of the whole lung. Moreover, a whole lung 3D analysis may result in less overestimation of hyperinflation. (D) Another limiting factor might be the duration of the inspiratory pause used in this study (4 s). According to David et al., pauses longer than 4 s could promote alveolar recruitment (46). Moreover, some studies indicate that an inspiratory pause shorter than 10 s when examining tomographic slice areas may overestimate alveolar recruitment due to overinflation without accounting for the accumulation of gas over the wells, providing the same estimates as in regions that are not gravity-dependent (47). (E) The absence of CT measurement at zero airway pressure during disconnection of the patient from ventilator could be another limitation, as the overinflated lung area at 5 cmH_2_O inspiratory pressure cannot be compared to a non-pressurized condition and the 2.1% of overinflated lung could be a representation of voxels within the large airways. (F) Finally, the time spent in each inspiratory pressure might be considered too short, evidenced mainly by differences in PaCO_2_ values during inspiratory pressure stepwise increase and decrease. Probably with more time to equilibrate CO2 washout, these values would be more similar at the same inspiratory pressure level. Moreover, it is known that a lack of PEEP is related to the appearance of collapsed areas and acute lung injury by cyclic opening and closing of the alveolar units, and increasing time at each pressure level, we could expect greater areas of the non-aerated lung. However, a longer time at each inspiratory pressure would greatly increase the total time of anesthesia in each animal, as well as the risk of hemodynamic instability.

In conclusion, increases in airway pressure beyond 5 cmH_2_O cause a progressive overinflation of the lungs that can encompass 28.4% of the parenchyma at 15 cmH2O. Ventilation with low driving pressures and with adequate respiratory rate may provide the best ventilatory strategy to small animals with highly compliant chest walls.

## Data Availability Statement

The original contributions presented in the study are included in the article/supplementary material, further inquiries can be directed to the corresponding author.

## Ethics Statement

The animal study was reviewed and approved by Ethics Committees for Animal Research at the Faculty of Veterinary Medicine and Animal Science of the University of São Paulo (FMVZ—USP Protocol Number 110/8) and Faculty of Medicina of the University of São Paulo (CEUA—USP protocol number 100/10). Written informed consent was obtained from the owners for the participation of their animals in this study.

## Author Contributions

AM: study design, data acquisition, analysis and interpretation of data, and drafted the manuscript. AA, DF, DO, and LM: conceived the study, study design, advice on data analysis and interpretation, and manuscript revision. AP and LV-M: study design and data acquisition. JS: revised the manuscript. All authors contributed substantially to manuscript revision and approved the final manuscript.

## Conflict of Interest

The authors declare that the research was conducted in the absence of any commercial or financial relationships that could be construed as a potential conflict of interest.

## Publisher's Note

All claims expressed in this article are solely those of the authors and do not necessarily represent those of their affiliated organizations, or those of the publisher, the editors and the reviewers. Any product that may be evaluated in this article, or claim that may be made by its manufacturer, is not guaranteed or endorsed by the publisher.
